# ‘Life is still worth living’: a pilot exploration of self-reported resources of palliative care patients

**DOI:** 10.1186/s12875-016-0450-y

**Published:** 2016-05-10

**Authors:** Franca Warmenhoven, Peter Lucassen, Mieke Vermandere, Bert Aertgeerts, Chris van Weel, Kris Vissers, Judith Prins

**Affiliations:** Department of Primary and Community Care, Radboud University Nijmegen Medical Centre, Huispost 117, Postbus 9101, 6500 HB Nijmegen, The Netherlands; Academic Centre for General Practice KU Leuven, Kapucijnenvoer 33, Blok j, bus 7001, B3000 Leuven, Belgium; Department of Anesthesiology, Pain and Palliative Medicine, Radboud University Nijmegen Medical Centre, Huispost 717, Postbus 9101, 6500 HB Nijmegen, The Netherlands; Department of Medical Psychology, Radboud University Nijmegen Medical Centre, Huispost 840, Postbus 9101, 6500 HB Nijmegen, The Netherlands

**Keywords:** Palliative care, Depression, Mood symptoms, Psychological adaptation, Psychological resilience

## Abstract

**Background:**

Facing a terminal illness can be highly stressful and palliative care patients frequently suffer from mood symptoms. The focus of health care is often on treating symptoms whereas health-promoting factors receive less attention. The aim of this study was to explore the views of palliative care patients on resources and ways of coping that help them prevent or manage mood symptoms.

**Methods:**

A pilot qualitative study was performed through face-to-face semi-structured interviews with fifteen ambulant patients with advanced cancer. The interviews were transcribed verbatim and qualitative analysis was performed independently by two researchers, according to the principle of constant comparative analysis.

**Results:**

Patients reported on attitudes and specific coping strategies that they found helpful, as well as aspects of their life narrative and spirituality. Resources were found in meaningful contacts with family and friends and in personal attention of professional medical caregivers for their wellbeing.

**Conclusions:**

We conclude that palliative care patients could identify resources to cope with mood symptoms in the context of their unique life. In helping patients to identify the personal resources that are accessible and available in their specific context, patient autonomy in enhancing resilience could be increased.

## Background

Living with a terminal illness is a highly stressful situation, causing substantial impact on the way patients relate to their social network and their way of living [1]. Patients in palliative care frequently report a variety of physical and psychological symptoms including depressive symptoms [2]. In the light of symptom control, palliative care should address and treat these symptoms.

Some, however, consider depressive symptoms a normal reaction to ‘something bad happening’ [3]. In this latter view, the absence of a depressive symptoms, or even reports of positive mood or emotions, in the context of a terminal illness are perhaps more surprising than the presence of depressive symptoms. Research points to positive emotions that may broaden an individual’s repertoire in reaction to adverse events and reinforce an individual’s personal resources [4]. A recent study confirmed this theory and suggested that upward spirals of positive emotions can counter downward spirals of negativity [5]. Antonovsky emphasized origins of health in a model that identifies three foundations for successful coping: comprehensibility, manageability and meaningfulness [6]. Whereas our healthcare system emphasizes the treatment of diseases and symptoms, a growing field of research is reflecting on ways to enhance health-promoting factors, inferring that these are also important in preventing disorders [7].

The aim of palliative care is to improve the quality of life of the individual patient by proactive identification of problems and delivering patient-centered multidimensional care [8]. It has been shown that adequate coping strategies can positively influence mood in palliative care patients [9], but little attention has thus far been given to palliative care patients’ actual experience of effective ways of coping and resources. This hampers the possibility to build palliative care upon self-empowerment. Preventive, context-related interventions such as those derived from positive psychology [10], can empower patients in a palliative care trajectory to rely on personal resources and ways of coping when facing terminal illness.

This exploratory study analyses the views of patients in a palliative trajectory on resources and ways of coping that help them prevent or manage mood symptoms in facing terminal illness and end of life decisions.

## Methods

A qualitative study was performed through face-to-face semi-structured interviews with patients with advanced cancer in a palliative care trajectory.

This study was part of a larger study in which 65 palliative care patients were included in a diagnostic study on depressive disorder [11]. Patients were recruited from an academic oncology and palliative care outpatient clinic and from different family practices that were affiliated with the Radboud University Nijmegen Medical Centre. All patients suffered from advanced cancer and had an estimated life expectancy between two months and one year according to their treating physician. Patients who were unable to speak Dutch or who suffered from cognitive dysfunction were excluded from this study. Part of the diagnostic study consisted of a face-to-face psychiatric interview (Schedules for Clinical Assessment in Neuropsychiatry 2.1 (SCAN 2.1)) to assess a possible depressive disorder. The interviews took place at the patients’ homes. First, an assessment of depression and anxiety was performed through a structured psychiatric interview, the SCAN 2.1. After this structured interview, which lasted between 15 min and 45 min, the open question ‘What helps you to prevent or address mood symptoms in the context of your terminal illness?’ was posed after which resources and ways of coping that helped the patient to prevent or manage mood symptoms in the context of facing terminal illness were explored in an open conversation. The interviews were recorded on audiotape. The interviews took place from December 2008 until November 2009. The current qualitative study reports about individual interviews with palliative care patients. For this pilot study fifteen palliative care patients were selected using purposive sampling based on age, sex and score on the Beck Depression Inventory (BDI-II). None of the patients suffered from a depressive disorder as assessed by the SCAN 2.1, but because the depression symptom burden can vary greatly, even in non-depressed patients [11], we selected about half of the patients who had a BDI-II depression score above a cut-off score of 16, and the rest of the patients who had scores below the cut-off score [12].

The interviews were transcribed verbatim and qualitative analysis of data has been independently performed according to the principle of constant comparative analysis [13, 14] by the interviewer and a second researcher. In the thematic analysis, both researchers coded the transcripts and categorized relevant and meaningful fragments with the aim of creating structure in relevant themes. This process was carried out using ATLAS.ti 4.2, a software program for qualitative data analysis. After analyzing seven interviews, the emerging codes and themes were discussed between the researchers until consensus was reached. The following eight interviews were analyzed using the codes and themes that had emerged from the first seven interviews; also new codes or themes were added when needed. During the qualitative analysis, themes were continuously checked against the transcripts to ground the themes in the data. No new codes emerged in the last two interviews.

## Results

Eight women and seven men participated in this study. Patients had a mean age of 65 years with a range of 51-89 years. On inclusion patients had a mean score on the BDI of 15.9, with a range from 5-35 (Table 1). None of the patients were suffering from a depressive disorder or anxiety disorder as assessed with the diagnostic interview. Three men and four women reported a history of depression. The median time that had passed since receiving the bad news of having an incurable illness was 22 months, with a range from 1-115 months.

**Table 1:** Patient characteristics

	Men	Women	Total
N	7	8	15
Age (mean and range)	66 (range 51-89)	65 (range 52-75)	65 (range 51-89)
BDI (mean and range)	15.3 (range 6-35)	16.5 (range 5-26)	15.9 (range 5-35)
Self-reported history of depression	*N* = 3	*N* = 4	*N* = 7
Months since receiving diagnosis of incurable illness (median and range)	27 (range 2-114)	9 (range 1-115)	22 (range 1-115)

Patients named resources or ways of coping that they experienced as helpful in preventing or addressing mood symptoms. In the answers to the question ‘What helps you to prevent or address mood symptoms in facing your terminal illness?’, we could distinguish three categories of resources: firstly, the resources and ways of coping that were directly related to the patient, secondly, the resources that were related to the patient’s social network and thirdly, the resources that were related to the professional support that patients received. An overview of the codes and themes that emerged in these three categories is presented in Table 2.

**Table 2:** Codes and themes emerging from the interviews

Category	Theme	Code
Resources related to the patient	Personal attitude	Optimistic nature or character
		A stance of acceptance or surrender
		Being opinionated
		Having a fighting spirit
		Enjoying
		Being able to be alone
		Humour
		Life setting
		Having no fear for deterioration or death
		Seeing the positive side of things
		Being self reflective
	Coping	Actively solving problems
		Engaging in activities
		Finding alternatives, being flexible with limitations
		Not thinking about the future
		Using effective coping skills from the past
		Different coping skills in different phases of the illness trajectory
		Making jokes
		Not being busy with illness all the time
		Distraction
		Self care
	Spirituality	Thoughts about the hereafter
		Belief in a higher power
		Having been raised in a religious tradition
		Not having a belief
		Religious rituals
		Attitude towards life
Resources related to social network	Family	Connection with children and grandchildren
		Not leaving family members behind
		Support from partner/spouse
	Social surroundings	Deeper connection with others
		Feeling part of a larger community
		Reactions from others
		Support
Resources related to professional support	Health care professionals	Health care professionals who actively ask about the patient’s well-being
		Professional care

### Resources related to the patient

In the reported resources that were directly related to the patient, three themes could be distinguished. Firstly, patients reported personal attitudes that they experienced as helpful, secondly, they referred to specific ways of coping that they used and thirdly, they reported resources that were of a spiritual nature.

Some personal attitudes that patients reported were related to their personal character, such as an optimistic nature or character, being opinionated and able to take decisions or having a fighting spirit but also a meekness or acceptance to the inevitable.

P. 4: ‘Everything I can do, that I can still enjoy, I do.’

P. 8: ‘So it is not only negative. I don’t see it that way. There are, of course, many negative things about it, but yes, life is still very much worth living.’

P. 14: ‘It is a surrender. To life, or destiny. I don’t have to fulfill that anymore’

Other personal attitudes had to do with their life narrative and how they looked back on their life. Patients reported about how their life narrative helped them to face their terminal illness. They reported experiences and memories from the past that helped them in the situation that they currently faced. Furthermore, patients reported that it was helpful that they could look back at their life with satisfaction and without the sense of leaving unfinished business behind.

P. 4: ‘…for a while, after I did not have to work anymore, I have been helping out as a volunteer in supporting disabled persons on their holiday. And then you actually learn a lot…..that there are other people and things can be different…….’

P. 11: ‘I always loved to go to work. Very much so. I have always worked very hard. I did not make weeks of forty hours, but I [started] in the morning at three-four am and I returned home at ten pm. On Friday at five am and Saturday afternoon home at three pm. Then I did not see my bed at all, at the bakery. It was always very nice work.’

P. 12: ‘I have had a very nice life. So, for me….well of course there are always things of which you could say: I would have liked it better this way or that way……but I have had a very nice life and…..a good marriage….not without any fight, I mean, there were also…..sometimes there were problems, but that was never paramount…’

P. 12: ‘Well, I have the idea, but again, you never know, that there are no unfinished things….I do not have to think: this needs to be…..with this person I need to reconcile……so yes, things are ok….I am at peace.’

Identified as a second theme within the resources related to the patient, patients reported specific ways of coping, such as: active problem solving, living with their limitations in a flexible way, distracting themselves, taking good care of themselves, undertaking various (physical) activities and living life as normal as possible. Patients also reported that they used ways of dealing with difficulties that had been proven effective in their past experiences and at different times in the illness trajectory, different ways of dealing with difficulties were used.

P. 4: ‘I don’t give in. I, yes, actually, I still try to do everything. ………normally [this activity] takes ten minutes. Well, it takes me one hour. …..And I could say that it used to take me only a little while, and now every time it takes me longer than back then. But I am happy and thrilled that I can still do it, that I am still able…..’

P. 8: ‘I love to enjoy nature and those kinds of things. …..We [patient and partner] did a bird course together for example. Our children thought it was deeply insane [laughs], but we think it’s incredibly fun and if we see a woodpecker when we take a walk, then we are completely happy [laughs].’ The third theme identified in the data regarding resources related to the patient was spirituality. Spirituality was experienced in religious rituals by some patients and in non-religious experiences (e.g. nature or connectedness with other people) by others. Active awareness of spirituality helped patients to feel connected with something larger. They felt they could surrender to something higher and sometimes participated in supporting rituals.

P.1 : ‘I am, yes, every human being is religious, you could say, but…..my view on life is just…..you come at a certain moment and you don’t choose this yourself. And you go at a certain moment and you don’t choose that either.’

P. 15: ‘I pray twice a day. For all the people that have died.’

### Resources related to social network

In the resources that were related to the social network of the patient, two themes could be identified: resources related to family and resources related to the patient’s social surroundings. Patients with a partner experienced the care of their partner as essential and the contact with family members was reported to be important.

P. 15: ‘I have a daughter who came to the radiation therapy 32 times. All these days.…. That’s positive….I am very happy about that’.

Furthermore, a sense of connectedness with a larger community (family, church, village) was reported to be helpful. Some patients reported that communication with other people was very supporting. They actively looked for meaningful contacts and interactions. Explicitly being of significance to other people was reported to be important.

P. 14: ‘If it becomes too much for me, I look for someone to talk with, for a while. So I can….that is…how shall I say this…in my own environment I cannot share much with people, except for my friends and daughter. So then…. I used to go to a course and there I would always find my kind of people. And now I have found someone to talk to through an organization of volunteers…… he used to be a priest. He is married and very involved in Buddhism. Yes, then you can speak with each other for a while and that brings something different…….yes, for example we exchange books, and then we talk about it.’

P. 9: ‘That’s the most important thing in life, isn’t it, that I am still useful for people and that the people that live around me are still useful to me’.

### Resources related to professional support

Patients valued a genuine interest of their professional caregivers in their wellbeing. Some reported that professional psychological care had been helpful. Patients also reported that it would be helpful if medical caregivers, like physicians and nurses, would more frequently ask about their wellbeing.

P. 9: ‘I went to Mrs. A [a psychologist], because I had difficulty communicating with my daughter because we have such conflicting opinions and I thought: we must not let this happen. So let’s find help.’

P. 11: ‘….the doctor will probably walk in this evening, he always comes walking in and out, once a week. …….we drink coffee and we talk for a while…..that is really great.’

P. 9: ‘It would be nice if something was offered. And maybe not specifically, but if they would, for example, if the specialist checks my breathing, checks whether my abdomen hurts etc., she could also ask if I am still feeling happy. Or if I am, I don’t know, crying more, or something like that……because that is not what she is asking. It would be nice if that was asked, because then you at least have an opportunity to talk about it. It could well be that there is no-one at home talking about it with you.’

## Discussion

This study reports self-reported resources and ways of coping that help patients to prevent or manage mood symptoms in facing terminal illness using qualitative research methods. Patients reported about attitudes and specific coping strategies that they found helpful, as well as aspects of their life narrative and spirituality. Furthermore, resources were found in meaningful contacts with family and friends and in personal attention of professional medical caregivers for their wellbeing.

Some of the self-reported resources that were identified in this study have been studied previously. Both an accepting stance towards the situation as well as an active attitude, as reported by patients in this study, have been found by Laarhoven et al. as being predictive for a better quality of life and lower levels of depression and hopelessness [9]. A review concluded that active, problem-focused coping in advanced cancer patients seems adaptive [15] which may be related to the ‘active stance towards life’ that patients in this study identify as being helpful. This quality of an ‘active stance towards life’ may also be related to the findings in a study in cancer patients in which goal reengagement, active coping and acceptance were reported to be significantly related to positive change [16]. In previous studies, practicing and experiencing religious rituals or spirituality seemed to be beneficial for quality of life in the context of palliative care [17, 18]. Earlier studies on social resources showed that a lack of social support was a predictor of non-remission for depression [19].

Every palliative care patient experiences his symptoms or moods in the context of his unique life and therefore seems the most relevant person to identify personal resources. Previous research shows that physicians can help patients with medical problems to identify and mobilize self-assessed personal health resources [20–22]. Considering this, palliative care patients could be given an active role in the care team in addressing, activating and enhancing personal resources to address mood symptoms. This could enhance their autonomy in creating or enhancing upward spirals of emotions and experience positive affect, which may be much more effective to counter the downward spiral of emotions that seem inevitable in the palliative care context [4].

In this study a differentiated sample of patients was used, including men and women in a palliative trajectory, varying in age, depression score on the BDI-II, history of depression and time since the diagnosis of an incurable illness. Each researcher performed the analysis independently. No cyclic analysis procedure was used, however, which makes it possible that themes were missed. A further limitation of this study is that it is not clear whether patients verbally identifying resources can also use and enhance these resources and whether the personal resources are effective in alleviating mood symptoms.

## Conclusions

In this study palliative care patients seem to welcome active exploration of their mood by healthcare professionals and can identify their personal resources to prevent or address mood symptoms. Although negative aspects of mood, such as symptoms and behavior of sadness or grief, are expected in a palliative care context, the findings of this study encourage also attending to positive experiences and behavior of patients in a palliative care setting. Palliative care patients can be encouraged to identify personal resources that are accessible and available in their specific context. The search for positive resources could offer a more inclusive and realistic view of the palliative care context in which both negative and positive aspects are attended to. In helping patients to identify their personal resources, patient autonomy in enhancing resilience could be increased. In this process it is essential that healthcare professionals invite patients as partners in the communal process of providing palliative care. More robust research in this field is necessary, firstly to further explore patients’ experiences of resources that are of use in facing terminal illness and secondly to clarify the effectiveness of actively involving patients in identifying and strengthening personal resources. Possibly this could lead to specific interventions that can support patients in the end of life phase.

### Ethical approval and consent

Institutional approval of the medical ethical committee at the University Medical Centre Nijmegen was obtained. We confirm all patient/personal identifiers have been removed or disguised so the patient/person (s) described are not identifiable and cannot be identified through the details of the story.

### Consent for publication

Informed consent was obtained from all participants in this research project.

### Availability of data and materials

The authors have full control over the primary data and the data can be reviewed on request. The primary data can be found at the department of Anesthesiology, Pain and Palliative Medicine, Radboud University Nijmegen Medical Centre, huispost 717, Postbus 9101, 6500 HB Nijmegen, The Netherlands.

## Abbreviations

ATLAS.ti 4.2: archiv for technik, lebenswelt und alltagssprache–text interpretation
BDI-II: beck depression inventory
SCAN 2.1: schedules for clinical assessment in neuropsychiatry
